# Acculturation and Age Acceleration: Differences Between Hispanic and Non-Hispanic Immigrants

**DOI:** 10.1093/geroni/igaf122.3696

**Published:** 2025-12-31

**Authors:** Antonio Bustillo, M Maria Glymour, Kaylie Moropoulos, Katrina Kezios, Adina Zeki Al Hazzouri

**Affiliations:** Columbia University Mailman School of Public Health, New York, New York, United States; Boston University, Boston, Massachusetts, United States; Columbia University Medical Center, New York, New York, United States; Boston University, Boston, Massachusetts, United States; Columbia University Medical Center, New York, New York, United States

## Abstract

Acculturation by immigrants to the United States is tied to dissipating immigrant health advantages, but its relationship to epigenetic age acceleration (AA) has not been studied. Further, acculturation may affect Hispanic and non-Hispanic immigrants differently. We estimated the effects of acculturation on epigenetic AA by Hispanic ethnicity using 2nd & 3rd generation epigenetic age clocks from the Health and Retirement Study (N = 4014). AA scores were defined as standardized residuals from models regressing each clock on chronological age. Acculturation measures included immigration age (0-15 years,16-40, 41-90 vs. non-immigrant); time since immigration (0-39 years, 40+ vs. non-immigrant); immigration year (1923-1964, 1965-2009 vs. non-immigrant); and English spoken at home (yes, no vs. non-immigrant). We predicted mean AA scores per acculturation measure level from confounder-adjusted regression models separately for Hispanics and non-Hispanics; we computed contrasts & 95% confidence intervals (Contrast [95% CI]) between predicted means for each acculturation measure level vs. non-immigrants. Hispanic immigrants showed net age deceleration if they migrated between ages 0-15 (Levine/PhenoAge= -0.28 [-0.56, 0], migrated after 1965 (Levine/PhenoAge = -0.18 [-0.36, -0.01]); GrimAge = -0.17 [-0.32, -0.01]), and regardless of whether they spoke English at home (Levine/PhenoAge = -0.3 [-0.57, -0.04]; GrimAge = -0.16 [-0.31, 0]). Non-Hispanic immigrants showed net age acceleration if they migrated between ages 41-90 (Levine/PhenoAge=0.49 [0.14, 0.84]; DunedinPoAm38 = 0.33 [0.02, 0.65]); immigrated 0-39 years ago (Levine/PhenoAge = 0.26 [0.04, 0.49]); and if they migrated after 1965 (Levine/PhenoAge = 0.2 [0.02, 0.38]). Acculturation may lead to aging advantages for Hispanic immigrants, but disadvantage for non-Hispanics immigrants.

